# The Prospective Associations between Self-Efficacy and Depressive Symptoms from Early to Middle Adolescence: A Cross-Lagged Model

**DOI:** 10.1007/s10964-016-0614-z

**Published:** 2016-11-29

**Authors:** Yuli R. Tak, Steven M. Brunwasser, Anna Lichtwarck-Aschoff, Rutger C. M. E. Engels

**Affiliations:** 1 Radboud University Nijmegen, Behavioural Science Institute, Developmental Psychopathology, Montessorilaan 3, room A 06.18, 6525 HR Nijmegen, The Netherlands; 2 Division of Allergy, Pulmonary, and Critical Care Medicine, Vanderbilt University School of Medicine, T-1218 Medical Center North, 1161 21st Ave. S., Nashville, TN 37232-2650 USA; 3 Radboud University Nijmegen, Behavioural Science Institute, Developmental Psychopathology, Montessorilaan 3, room A 06.01, 6525 HR Nijmegen, The Netherlands; 4 Department of Developmental Psychology, Utrecht University, Martinus J. Langeveldgebouw, Heidelberglaan 1, 3584 CS Utrecht, The Netherlands; 5 Trimbos-institute, Da Costakade 45, 3521 VS Utrecht, The Netherlands

**Keywords:** Depressive symptoms, Self-efficacy, Adolescence

## Abstract

Over the course of adolescence, an increasing number of adolescents experience depression. In order to effectively target depression, identifying risk factors for depressive symptoms is pivotal. Since low levels of self-efficacy were associated with higher levels of depressive symptoms in previous studies, the current study investigated the bidirectional and prospective associations between depressive symptoms and academic, social and emotional self-efficacy from early to mid adolescence in a cross-lagged path model. The sample consisted of 1,341 adolescents (47 % girls) with a mean age of 14 years, SD = 0.56. Depressive symptoms and self-efficacy levels were assessed every 6 months over a period of 2.5 years. Depressive symptoms predicted subsequent levels of academic and emotional self-efficacy on all time points, and social self-efficacy on one time point. Self-efficacy did not predict subsequent levels of depressive symptoms. There was no evidence of sex differences in the cross-lagged associations between depressive symptoms and self-efficacy levels. Implications of the findings are discussed.

## Introduction

Depression is one of the leading causes of disability worldwide (WHO 2008). During adolescence, the lifetime prevalence of depressive disorders increases, from 1.1 % at age 11–20.7 % at age 18 (Thapar et al. 2012). In the Netherlands, between 9–21 % of adolescents report depressive symptoms, with girls outnumbering boys, ratio 2:1 (Wijga et al. 2010). Experiencing elevated depressive symptoms during adolescence is associated with increased risk for future mood disorders (Roza et al. 2003), and suicide (Bridge et al. 2006). Therefore, prevention and treatment of depression is urgently needed (WHO 2013). In order to design effective prevention programs for adolescent depression, research exploring the way in which depressive symptoms develop during adolescence is pivotal.

During adolescence, youngsters face challenges in the social, emotional and academic domains. Their social world changes radically as peers and friends become a major source of support, romantic relationships emerge and their relationship with parents undergoes important transitions (Collins 2003; Steinberg 2001). At the same time adolescents face more stressful life events compared to children, but the cognitive capacities and the emotion regulation strategies to cope with these stressors are still developing during adolescence (Steinberg 2005b). In addition, academic development and achievement is an important developmental task with which many adolescents struggle (Steinberg 2005a).

### Self-Efficacy

A crucial factor for adolescents’ emotional well-being is their belief in their own capacities to face those challenges. In the literature, these beliefs have been defined as *self-efficacy* beliefs. According to Bandura’s social cognitive theory (Bandura 1977), self-efficacy is the belief in one’s own abilities to perform the actions that are needed to obtain a desired goal. Self-efficacy beliefs guide behavior both directly, and indirectly through personal goals, expectations regarding the outcome of certain behavior, and environmental influences (Bandura 2012). Social cognitive theory reveals several possible pathways through which self-efficacy can be acquired (Bandura 1998, 2012). First, self-efficacy builds through overcoming obstacles and experiencing success, success that depends on a person’s own actions or contributions. Second, seeing similar others overcome obstacles and attain their goals may also contribute to self-efficacy. Third, self-efficacy can be promoted through social persuasion in which others tell a person that he or she is competent. Importantly, self-efficacy is fairly independent of a person’s actual skills but individuals with high self-efficacy beliefs show more perseverance when facing obstacles (Bandura 2012).

It has been argued that self-efficacy beliefs may vary across contexts (Pastorelli et al. 2001). That is, a person may experience different levels of self-efficacy across various domains, he/she might have a high level of self-efficacy with respect to academic functioning, but a low level of self-efficacy in the social domain. Adolescents who report depressive symptoms often show impairments in academic, social and emotional functioning concurrently and several months later (Jaycox et al. 2009). They often experience concentration problems (Wesselhoeft et al. 2013), have less academic and occupational aspirations (Gotlib et al. 1995), obtain lower grades and have lower levels of academic self-efficacy (Jaycox et al. 2009). Moreover, adolescents who suffer from depressive symptoms do not function adequately with peers and feel less supported by their peers and parents and have more conflicts with parents (Jaycox et al. 2009). In addition, experiencing depressive symptoms is often associated with the use of less effective and adaptive emotion regulation strategies such as avoidance, rumination and suppression (see for a review Aldao et al. 2010). Finally, these adolescents report a higher number of impaired days and a lower quality of life (Gotlib et al. 1995; Jaycox et al. 2009).

Since adolescents who report depressive symptoms show impairments in the academic, social and emotional domain, the current study focuses on academic, social and emotional self-efficacy, based on the work by Muris (2001). *Academic self-efficacy* refers to the degree to which a person feels he/she is able to fulfill academic expectations and to cope with academic challenges such as finishing homework on time, preparing for a test, and passing exams. *Social self-efficacy* is the belief in one’s ability to become and stay friends, be assertive, function adequately in the peer context, and approach unfamiliar persons. *Emotional self-efficacy* is the belief in the ability to regulate and control negative emotions and thoughts, to cheer oneself up and calm oneself down.

### Bidirectional Association between Self-Efficacy and Depressive Symptoms

Self-efficacy might show a bidirectional association with depressive symptoms. This idea is supported by the stress generation theory of depression (Hammen 2006) and the socio-cognitive theory of Bandura (2012). In the stress generation theory of depression (Hammen 2005, 2006), it is described that people who experience depressive symptoms generate more interpersonal stressors compared to healthy people. Both individual characteristics, such as dysfunctional social problem solving skills, cognitions and beliefs, as well as negative environments, such as a violent spouse or being poor, contribute to interpersonal stress (Hammen 2005, 2006). For example, it has been found that low levels of effortful engagement and high levels of disengagement lead to an increase in interpersonal problems (Flynn and Rudolph 2011). When encountering interpersonal stressors, people feel that they lack the abilities to cope with those stressors and to solve the interpersonal problems. In other words, people have lower levels of emotional and social self-efficacy, which in turn could lead to an increase in depressive symptoms (Rudolph et al. 2008).

Social cognitive theory argues that a lack of self-efficacy might lead to feelings of depression through a discrepancy in aspirations and perceived skills. Adolescents feel they lack the ability to attain their standards, but at the same time think they should be able to do so (Bandura et al. 1999). Often, those standards are set unrealistically high. Because of this discrepancy, adolescents might be less likely to perform actions to obtain their goals, which could further negatively impact their self-efficacy by means of negative self-talk. This negative self-talk and low levels of self-efficacy might in turn increase the level of depressive symptoms.

On the other hand, it is argued that poor emotional well-being, as indicated by feelings of depression and anxiety, might impede self-efficacy beliefs (Bandura 1977). As described above, adolescents suffering from depressive symptoms often show less effective emotion regulation such as avoidance, rumination and suppression (Aldao et al. 2010). These emotion regulation strategies are known to increase negative mood and anxiety (Carver and Connor-Smith 2010), which might result in even lower levels of emotional self-efficacy. In addition, depression is associated with impaired functioning in social contexts (peers and family; Jaycox et al. 2009). Depressed adolescents often withdraw from social contacts and interactions (Vargo 1996), resulting in fewer (positive) peer interactions and friends (Schaefer et al. 2011), which might undermine their feeling of social self-efficacy. Finally, high depressive symptoms are associated with impaired academic functioning (Jaycox et al. 2009). Depressed adolescents show concentration problems (Gotlib and Joormann 2010), which might result in lower grades and more academic failing (Darney et al. 2013), and could therefore also lead to lower levels of academic self-efficacy.

Research shows that low levels of self-efficacy are concurrently associated with depressive symptoms for adults (Bandura 1997), adolescents (e.g., Bandura et al. 2003), and children (Bandura et al. 1999; Steca et al. 2014) across various countries around the world (Luszczynska et al. 2005). In adolescent samples, concurrent associations were found between self-efficacy and depressive symptoms, as low levels of academic (Bandura et al. 1996, 2003, 1999; Muris 2001, 2002; Muris et al. 2015), and emotional self-efficacy (Caprara et al. 2010; Garber et al. 1995; Muris 2001, 2002; Muris et al. 2015) were associated with higher levels of depressive symptoms. Evidence of an association between social self-efficacy and depressive symptoms in adolescence has been less consistent. Whereas at least two studies reported a negative concurrent association (Bandura et al. 1996, 1999), another study reported no association (Muris et al. 2015).

Longitudinal studies on the association between self-efficacy and depressive symptoms are less frequently conducted. However, higher levels of emotional self-efficacy lead to lower levels of depressive symptoms at 2 years follow-up in middle adolescence (Bandura et al. 2003). Recently, it was reported that only academic self-efficacy, and not social self-efficacy, predicted depressive symptoms at 6 to 8 months follow-up in adolescents from fifth to eight grade, when controlling for shared variance of academic and social self-efficacy (Scott and Dearing 2012). Research into the prospective association of depressive symptoms to self-efficacy is less frequently conducted. To our knowledge, only one study examined the prospective association of depressive symptoms on self-efficacy levels in adolescence. Adolescents experiencing higher levels of depressive symptoms reported lower levels of academic self-efficacy up to 6 months follow-up (Jaycox et al. 2009). Since adolescence is characterized by changes in the emotional, social and academic domain, which can impact emotional well-being (Steinberg 2005b), it is important to assess whether the association between depressive symptoms and subsequent academic, social and emotional self-efficacy levels show the same pattern in early compared to middle adolescence.

Girls and boys tend to differ in the extent to which they experience depressive symptoms and the pathways that lead to depressive symptoms (Hankin et al. 2007). It has been found that girls tend to report more depressive symptoms around the age of 13 compared to boys (Twenge and Nolen-Hoeksema 2002). In addition, some studies show that boys and girls differ in their level of self-efficacy. In general, boys report higher levels of self-efficacy compared to girls (Bandura et al. 2003; Caprara et al. 2010; Muris et al. 2015). Yet, girls report higher levels of academic self-efficacy (Bandura et al. 2003, 1999). Moreover, the association between self-efficacy and depressive symptoms was found to differ between boys and girls since low levels of social self-efficacy were associated with higher concurrent levels of depressive symptoms for girls, but not for boys (Bandura et al. 1999). To conclude, it is important to study whether there exist gender differences in the associations between depressive symptoms and self-efficacy levels during adolescence.

## The Current Study

Since it is argued that self-efficacy and depressive symptoms might influence each other over time, the current study examined the longitudinal and bidirectional associations between depressive symptoms and academic, social and emotional self-efficacy in a large sample spanning early to middle adolescence. We hypothesized that low levels of academic, social, and emotional self-efficacy would predict higher levels of depressive symptoms assessed 6 months later. Further, it was expected that higher levels of depressive symptoms would predict lower levels of self-efficacy in all three domains measured 6 months later. It was tested whether these associations remained constant during the 2.5 years of the study. In addition, it was examined whether boys and girls differed in the associations between depressive symptoms and the three self-efficacy domains. Since only few studies have been conducted regarding gender differences in relation to self-efficacy levels and regarding the association between self-efficacy and depressive symptoms in early compared to middle adolescence, no specific hypotheses were formed.

## Method

### Participants

The sample consisted of 1,341 Dutch adolescents, 634 girls with a mean age of 13.90 (SD = .49, Range = 12.26–16.35), and 707 boys with a mean age of 14.00 (SD = 0.56, Range = 11.58–16.00). Most adolescents (64.5 %) went to school in urban areas. High school students in the Netherlands are grouped into different educational tracks. In this study, 7 % were in the pre-vocational secondary educational track (PVSE; Dutch translation is VMBO), 51.2 % were in the higher general secondary educational track (HGSE; HAVO), and 41.8 % were in the pre-university educational track (PUE; VWO) (Dutch Ministry of Education 2014). Of the adolescents 83.1 % (*n* = 1,115) were of Dutch nationality, based on the definition that he or she and both parents were born in the Netherlands (Centraal Bureau voor de Statistiek 2016).

### Procedure

The current sample was drawn from a large depression prevention study (Tak et al. 2016). This study had a randomized controlled design and the prevention program was based on cognitive behavioral therapy aimed to reduce depressive symptoms, to improve self-efficacy, coping, optimism and life-satisfaction. To control for effects of the intervention, treatment condition was included as a covariate, as will be explained in the strategy of analyses. The participating adolescents were drawn from nine schools and were included through passive consent but could withdraw study participation at any time. Adolescents completed an online or paper assessment every 6 months over the course of 2.5 years. The six assessments were completed at school during school time. The adolescents who were absent during the assessments were asked to complete the questionnaire at home. Only the adolescents who completed the questionnaire outside school hours received a gift voucher of €7.50. At the third assessment five gift vouchers of €20 were distributed randomly to increase the participation rate of the adolescents who were not present at the initial assessment during school time. All participating adolescents at the last assessment received a gift voucher of €7.50. The ethics committee of the Faculty of Social Sciences at local university approved the trial design and research protocol, as registered by the Dutch Trial Registration. Attrition was low across assessments since 96.5, 89.4, 89.3, 83.7., 77.4, and 84.5 % of participants completed the questionnaire respectively at baseline and follow-ups.

### Measures

#### Depressive symptoms

Depressive symptoms were measured with the Dutch translation of the Children’s Depression Inventory (CDI) (Kovacs 1985; Timbremont et al. 2008). This is a reliable and valid measure of depressive symptoms (Evers et al. 2009–2011). For each of the 27 items adolescents had to indicate which of the three statements reflected their feelings best over the past 2 weeks. For example: “I am sad sometimes” (0), “I am often sad” (1), and “I am sad all the time” (2). Cronbach’s alpha was .84, .86, .87, .91, .91, and .89, at baseline and follow-ups, respectively. Item nine, which targets suicidal thoughts and ideation, was omitted from the questionnaire, due to ethical considerations. To correct sum depressive symptom scores for the missing item, the mean item score was multiplied by 27. Total scores could range from 0–54, and higher scores indicated a higher level of depressive symptoms.

#### Self-efficacy

The Self-Efficacy Questionnaire for Children (SEQ-C) consists of three scales: academic, social, and emotional self-efficacy (Muris 2001). The SEQ-C was found to assess self-efficacy reliably and validly in European samples (Kokkinos and Kipritsi 2012; Muris 2001, 2002), and in American adolescents (Suldo and Shaffer 2007). The SEQ-C was administered at the first five assessments up to 2 years follow-up. For each of the 21 items adolescents had to specify how well they thought they could perform the task described on a 5-point Likert scale ranging from 1 = not at all to 5 = very well. Academic self-efficacy consists of 7 items, for example, “How well can you study when there are other interesting things to do?” Cronbach’s alpha was .86, .88, .88, .90, and .88, at baseline and follow-ups respectively. Social self-efficacy consists of 7 items, “How well can you become friends with other children?” Cronbach’s alpha was .79, .87, .88, .91, and .88, at baseline and follow-ups respectively. Emotional self-efficacy consists of 7 items, for example, “How well can you prevent yourself from becoming nervous?” Cronbach’s alpha was .85, .88, .89, .91, and .89, at baseline and follow-ups respectively. Subscale sum scores ranged from 7–35. Higher scores indicated higher levels of self-efficacy.

### Strategy of Analyses

To test the longitudinal and bidirectional associations between depressive symptoms and the three self-efficacy domains, a cross-lagged path model (CLPM) was specified in Mplus 6.11 (Muthén and Muthén 1998–2010). CLPMs allow for the evaluation of reciprocal associations between multiple time-varying variables. In the typical CLPM with two time-varying response variables (Y and Z), Y at all time points (*t*) (with the exception of the first) is a function of: (1) scores on Y at the prior time point (autoregressive effect), (2) scores on Z at the prior time point (cross-lagged effect), (3) concurrent covariances among the response variables, and (4) model covariates. Similarly, scores on Z at all time points except the first are also a function of autoregressions, cross-lagged and concurrent associations with Y, and covariates. Scores on the response variables at time 1 are treated as exogenous.

Our general modeling approach was to begin with a highly constrained baseline model in which the autoregressive and cross-lagged effects, as well as the concurrent cross-variable correlations were held constant over time. We then specified more general models relaxing these constraints, allowing cross-lagged, autoregressive, and concurrent associations to vary in magnitude over time. A model specifying reciprocal associations among the three self-efficacy variables was fitted first before adding the reciprocal associations among the self-efficacy variables and depressive symptoms. The Satorra-Bentler scaled difference *χ*² test (Satorra and Bentler 2001) and the sample size adjusted BIC score were used to determine preference between competing models. Finally, multiple-group models for sex were specified testing whether the model fit was improved by allowing for separate parameter estimates (autoregressive effects, concurrent correlations, and cross-lagged associations) for boys and girls.

To assess the degree to which the assumption of independent errors was violated due to the clustering of students within schools, the intra-class correlation coefficient (ICC) was estimated. The mean ICC was .02 which indicates that only 2 % of the variance of depressive symptoms and self-efficacy can be attributed to clustering within schools. Therefore, we did not control for this small effect. To control for effects of the intervention and to control for age related differences in depression and self-efficacy, condition and age were included in the cross-lagged analyses as covariates. Parameters were estimated using robust maximum likelihood estimation (the MLR estimator in Mplus) which allows for the retention of participants with partial missing data on the dependent variables. MLR provides unbiased parameter estimates assuming that data are missing at random (MAR) (Muthén and Muthén 1998–2010).

## Results

The means and standard deviations of depressive symptoms and academic, social, and emotional self-efficacy for boys and girls are presented in Table 1. As can be seen, the level of depressive symptoms was low and rather stable over time. The percentage of adolescents scoring above the subclinical (CDI >= 13) and clinical (CDI >= 19) cut-off was calculated. In total 14.2, 17.7, 14.0, 18.2, 18.8, and 18.2 % scored above the subclinical cut-off score on assessment T1, T2, T3, T4, T5, and T6, respectively. In addition, 4.8, 7.4, 5.8, 10.3, 10.8, and 9.0 % scored above the clinical cut-off score on assessment T1, T2, T3, T4, T5, and T6, respectively. The level of self-efficacy was moderately high and comparable to the study by Muris (2001).

**Table 1:** Means and standard deviations of depressive symptoms, academic, social, and emotional self-efficacy

Variable		*M* (SD)	1	2	3	4	5	6	7	8	9	10	11	12	13	14	15	16	17	18	19	20	21
1	DS1	7.55 (5.70)	–																				
2	ASE1	24.53 (5.27)	−.55	–																			
3	SSE1	26.36 (4.32)	−.40	.26	–																		
4	ESE1	24.08 (5.18)	−.57	.35	.56	–																	
5	DS2	8.06 (6.59)	.59	−.38	−.23	−.35	–																
6	ASE2	24.82 (5.53)	−.38	.57	.12	.22	−.50	–															
7	SSE2	26.12 (5.08)	−.28	.17	.47	.31	−.42	.55	–														
8	ESE2	24.60 (5.35)	−.37	.20	.30	.51	−.50	.59	.72	–													
9	DS3	7.70 (6.33)	.53	−.35	−.28	−.33	.55	−.37	−.29	−.34	–												
10	ASE3	24.29 (5.33)	−.35	.52	.14	.21	−.37	.54	.25	.29	.51	–											
11	SSE3	26.03 (4.99)	−.27	.18	.47	.28	−.26	.25	.48	.34	−.43	.52	–										
12	ESE3	24.60 (5.17)	−.34	.20	.30	.46	−.35	.29	.35	.52	−.49	.56	.72	–									
13	DS4	8.56 (8.18)	.35	−.27	−.18	−.24	.37	−.26	−.21	−.24	.57	−.37	−.31	−.34	–								
14	ASE4	23.73 (5.73)	−.23	.39	.11	.12	−.29	.40	.19	.19	−.42	.56	.25	.25	−.48	–							
15	SSE4	25.06 (5.62	−.19	.16	.32	.17	−.23	.18	.33	.21	−.35	.26	.44	.30	−.46	.65	–						
16	ESE4	23.84 (5.62)	−.22	.16	.24	.33	−.30	.22	.28	.36	−.39	.30	.36	.47	−.48	.68	.80	–					
17	DS5	8.63 (8.15)	.37	−.29	−.20	−.23	.40	−.27	−.21	−.22	.47	−.36	−.27	−.30	.57	−.40	−.38	−.41	–				
18	ASE5	23.59 (5.44)	−.28	.44	.08	.15	−.29	.42	.14	.16	−.36	.54	.22	.22	−.36	.51	.27	.29	−.52	–			
19	SSE5	25.45 (5.09)	−.22	.21	.34	.20	−.22	.18	.32	.19	−.30	.25	.46	.27	−.32	.26	.46	.35	−.50	.56	–		
20	ESE5	23.87 (5.26)	−.27	.23	.20	.35	−.28	.21	.23	.34	−.32	.28	.31	.45	−.37	.27	.32	.47	−.54	.57	.72	–	
21	DS6	8.18 (7.44)	.39	−.29	−.19	−.26	.38	−.27	−.24	−.27	.50	−.33	−.31	−.35	.49	−.37	−.33	−.38	.61	−.39	−.35	−.38	–

*DS1 DS6* depressive symptoms time one—time six, *ASE1 ASE5* academic self-efficacy time one–time 5, *SSE1 SSE5* social self-efficacy time one—time five, *ESE1 ESE5* emotional self-efficacy time one—time five

All correlations were significant at the *p* < .01 level

When modeling the reciprocal effects of depressive symptoms and the self-efficacy domains, we first specified a model containing the three self-efficacy domains. In this model we controlled for age and condition by regressing them on academic, social and emotional self-efficacy. First, a constrained model was tested in which the stability estimates (autoregressions), and the cross-lagged paths of ASE, SSE, and ESE were set equal over time. The residuals for all self-efficacy domains were estimated and were allowed to correlate with each other. The concurrent correlations between the three self-efficacy domains were estimated. This model fitted the data well, *χ*² (66) = 302.690, *p* < .001, RMSEA = .039, 90 % CI = [.034–.044], CFI = .979, SRMR = .041, BIC = 28,570.929. Estimating the cross-lagged paths for all self-efficacy domains did not improve model fit, Satorra Bentler *χ*² (18) = 26.381, *p* = .091. In addition, the BIC-score were not improved, as the BIC increased to 28,667.748. Therefore, it was decided to keep the more parsimonious model in which the cross-lagged paths were constrained to be equal. In this model, all self-efficacy domains showed significant but moderate stability over time, β ranged between .10 and .18. In addition, all self-efficacy domains showed significant concurrent correlations with each other, r ranged between .26 and .84. However, the self-efficacy domains did not show any significant cross-lagged associations with other self-efficacy domains. This indicates that the self-efficacy domains did not predict levels of other self-efficacy domains on the next time point.

**Fig. 1:**
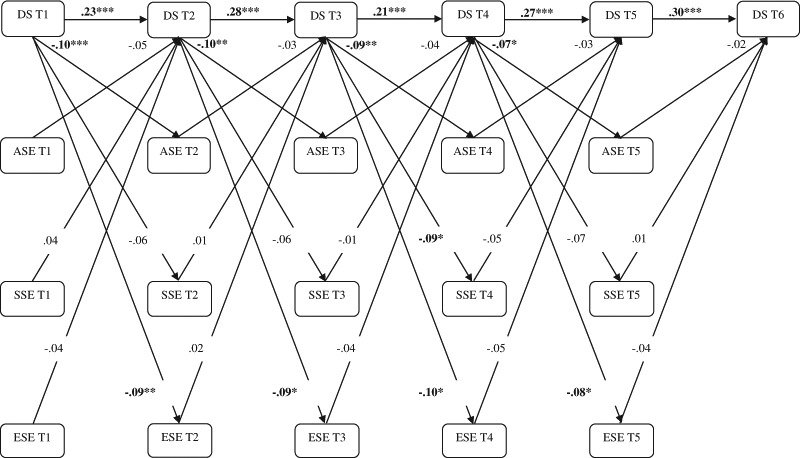
Cross-lagged model of depressive symptoms, academic, social and emotional self-efficacy (*N* = 1,341, standardized estimates). *DS* depressive symptoms, *ASE* academic self-efficacy, *SSE* social self-efficacy, *ESE* emotional self-efficacy. All other parameter estimates are presented in Appendix: the stability across time and the cross-lagged associations between SE domains, and the concurrent correlations. *p < 0.05; **p < 0.01; ***p < 0.001

To test the longitudinal cross-lagged associations between the self-efficacy domains and depressive symptoms, depressive symptoms were added to the final cross-lagged model of self-efficacy. The constrained model, in which the stability over time for depression and in which the concurrent correlations between depression and the self-efficacy domains were fixed over time, showed a good model fit, *χ*² (115) = 459.970, *p* < .001, RMSEA = .034, 90 % CI = [.030–.038], CFI = .977, SRMR = .043. In this model, both the residual variances and the cross-lagged paths were estimated. The second step was estimating the concurrent correlations between depression and the self-efficacy domains as well. This resulted in a significant model fit improvement, Satorra Bentler *χ*²Δ (12) = 46.241, *p* < .001, and the BIC scores decreased as well, 25,345.502 and 25,337.230, respectively. However, estimating the stability over time did not improve model fit, Satorra Bentler *χ*²Δ (4) = 6.474, *p* = .166, and the BIC score increased to 25,353.290. Therefore, in the final model the stability over time of depressive symptoms was fixed and the concurrent correlations between depression and the self-efficacy domains were estimated. This model showed an excellent model fit, *χ*² (127) = 402.656, *p* < .001, RMSEA = .032, 90 % CI = [.028–.036], CFI = .981; SRMR = .035. For parameter estimates, see Fig. 1 and Appendix table 2.

As can be seen in Fig. 1, depressive symptoms showed moderate stability over time. There were significant negative associations between depressive symptoms and levels of academic and emotional self-efficacy at the subsequent time point for each time lag. But depressive symptoms did not predict the level of social self-efficacy, except from T3 to T4. The self-efficacy domains did not predict subsequent levels of depressive symptoms.

To test whether the cross-lagged paths differed by sex, several multiple group models were run. First, allowing separate residual variances for sex improved model fit, *χ*²Δ (21) = 242.932, *p* < *.*001, and the BIC-score decreased as well. Second, allowing the concurrent correlations to vary between boys and girls as well, further improved model fit, Satorra Bentler *χ*²Δ (30) = 246.425, *p* < .001, and the BIC-score decreased also. Third, specifying separate autoregressions for sex, improved model fit even further, *χ*²Δ (4) = 29.092, *p* < .001, and the BIC-score was also lower. In general, the model explained more variance in depressive symptoms and self-efficacy for girls, compared to boys, as the R-square was larger for girls, and the residual variances were larger for boys. For girls, the stability in depressive symptoms and self-efficacy over time was higher compared to boys. Finally, allowing separate cross-lagged paths for sex improved model fit, Satorra Bentler *χ*²Δ (27) = 59.213, *p* < .001, RMSEA = .034, 90 % CI = [.030–.039], CFI = .978; SRMR = .048. However, the BIC score increased from 24,642.017 to 24,772.094, which indicates that the model fit has not improved. Because of our large sample size and the complicated model that was tested, even very small differences in the likelihood functions between the competing models would result in significant differences. We therefore can only conclude that there were compelling gender differences in the cross-lagged associations when all the fit indices would show a significant model fit improvement. Since the fit indices showed mixed results, we need to conclude that there were no differences in the cross-lagged associations between boys and girls.

## Discussion

Experiencing depressive symptoms and reporting low levels of self-efficacy has been found to be associated concurrently in adults (Bandura 1997), adolescents and children (Bandura et al. 2003; Steca et al. 2014). Only few studies have examined longitudinal associations in adolescence; from self-efficacy to depressive symptoms (e.g., Bandura et al. 2003; Scott and Dearing 2012), and from depressive symptoms to self-efficacy (Jaycox et al. 2009). Adolescence is a period of rapid and profound changes in several domains, including the social, emotional and academic domain (Steinberg 2005a). It can therefore be considered a sensitive developmental period in which adolescents develop confidence in their own ability to deal with social, emotional and academic challenges (i.e., sense of self-efficacy). At the same time, adolescence is a period in which mood disorders such as depression start to emerge (Kessler et al. 2005). Given those developments and the findings concerning the link between depressive symptoms and self-efficacy, this study was to our knowledge, the first to investigate the mutual influence between depressive symptoms and academic, social and emotional self-efficacy in a large adolescent sample, spanning 2.5 years over a period of early to mid adolescence.

The results showed that depressive symptom levels were negatively associated with academic and emotional self-efficacy consistently across four six-month time lags (2 years) when accounting for prior levels of self-efficacy and concurrent associations with depressive symptoms. However, the self-efficacy scales were not predictive of subsequent levels of depressive symptoms. Although autoregressive coefficients and residual variances tended to be smaller, and contemporaneous correlations between constructs larger, among girls than boys, there was not compelling evidence that the magnitude of cross-lagged associations differed by sex. That is, models holding the bidirectional longitudinal associations between depressive symptoms and self-efficacy constant across sex were preferable over those providing separate estimates for girls and boys.

The findings are in line with studies that describe concurrent and negative associations between self-efficacy and depressive symptoms in adolescence (Caprara et al. 2010; Muris 2001, 2002; Muris et al. 2015). In addition, and in line with expectations, the findings showed that higher levels of depressive symptoms predicted lower levels of subsequent academic and emotional self-efficacy for boys and girls. This might be explained by the findings that adolescents who experience depressive symptoms use less adaptive emotion regulation strategies (Aldao et al. 2010), and experience more academic difficulties (Jaycox et al. 2009). As a result, their emotional and academic self-efficacy might decrease. In contrast, depressive symptoms were not a strong predictor of social self-efficacy. Depressive symptoms were only predictive of subsequent levels of social self-efficacy from T3 to T4. It is unclear why that might be the case. During adolescence, brain regions and conceptions of relationships mature (Steinberg 2005b), and adolescents become more aware of the impact of their own behavior on their relationships. Therefore, adolescents might realize that their depressive behavior makes it more difficult to function well in social relationships. However, because depressive symptoms were not related to subsequent social self-efficacy levels consistently but just at one time point, other factors rather than depressive symptoms should be explored in the formation of adolescents’ sense of social self-efficacy. For example, acceptance from peers and closeness to parents are important predictors of adolescents’ global self-esteem (Skogbrott Birkeland et al. 2014), and might therefore also be important for the level of social self-efficacy. Alternatively, especially given that many associations were evaluated, the significant predictive association between depressive symptoms and social self-efficacy at this one time point may have been a chance finding.

Regarding the association of self-efficacy and depressive symptoms, it was found that self-efficacy did not predict subsequent levels of depressive symptoms. The finding that self-efficacy levels did not predict subsequent levels of depressive symptoms is in contrast to previous studies that found that emotional (Bandura et al. 2003) and academic self-efficacy (Scott and Dearing 2012) negatively predicted subsequent levels of depressive symptoms. This difference in findings might be explained by methodological differences between the current and previous studies. In the current study, the bidirectional associations between self-efficacy and depressive symptoms were modeled including the concurrent correlations and stability in depressive symptoms and self-efficacy domains over time. This model provides a detailed picture of the potentially complex associations between depressive symptoms and self-efficacy constructs. In previous studies, only the one-way prospective association of self-efficacy to depressive symptoms was assessed, sometimes controlling for previous levels of depressive symptoms. In these studies an important factor such as the level of stability in self-efficacy over time, might have been missed. Since the current study is one of the first studies assessing the bidirectional longitudinal associations between depressive symptoms and self-efficacy over time during early to middle adolescence using a cross-lagged model, more research is needed that study these bidirectional association during adolescence before firm conclusions can be drawn.

A second explanation for the current finding that depressive symptoms did not predict subsequent levels of self-efficacy domains, might be found in the fact that self-efficacy domains were rather unstable over time in the current study, i.e., previous academic self-efficacy was a significant but modest predictor of subsequent academic self-efficacy. First, this might indicate that other factors, as experiences of failure or success in the social, emotional and academic domain, are more important predictors of subsequent levels of social, emotional and academic self-efficacy than self-efficacy levels assessed 6 months ago. Second, this might also suggest that self-efficacy is still developing during early and middle adolescence. As described in the introduction, adolescents undergo changes in the cognitive, social, emotional and academic domain that might also impact their level of self-efficacy. A third explanation might be found in the observation that even in adults self-efficacy levels fluctuate depending on the events people experience (Yeo and Neal 2006). Self-efficacy levels might therefore be state dependent. Hence, when self-efficacy levels and depressive symptoms are measured on shorter time intervals, e.g., weeks, a predictive association of self-efficacy to depressive symptoms might be found. Ideally, the time interval of the study assessments corresponds to the speed in which the phenomenon that is studied develops naturally (van Geert and Lichtwarck-Aschoff 2005). Future studies should therefore investigate the association between depressive symptoms and self-efficacy levels during adolescence using shorter time intervals, e.g., weeks. In this way, our understanding of the development of self-efficacy and the changes in the association between self-efficacy and depressive symptoms might be improved.

In the current study, no sex differences were found: depressive symptoms predicted subsequent levels of self-efficacy, but not vice versa. However, the model as a whole explained more variance in depressive symptoms for girls compared to boys. This might suggest that different factors might be important in the development of depressive symptoms for boys vs. girls. The cognitive vulnerability-transactional stress model (Hankin and Abramson 2001) describes the onset of depressive symptoms in which different pathways to depressive symptoms might be identified for boys and girls. In this model, potential important factors and mechanisms are described for the development of depressive symptoms. It states that pre-existing vulnerabilities on different levels (genetic, personality and environment), affect cognitive vulnerabilities and the experience of negative life events. In addition, a diathesis-stress mechanism is described consisting of the interaction between negative cognitions, affect and life events, which results in an increase in depressive symptoms. Future studies could test hypotheses based on this model to improve our understanding of the development of adolescents’ depressive symptoms in both boys and girls.

### Strengths and Limitations

The current study extends prior research in several ways. First, the cross-lagged and bidirectional associations between self-efficacy and depressive symptoms were examined in one model, thereby controlling for the stability in depression and self-efficacy over time and the concurrent correlations between the two constructs. Previous studies mainly assessed the prospective association of self-efficacy to depressive symptoms. Second, three different aspects of self-efficacy were assessed, academic, social and emotional self-efficacy. Third, it was assessed whether there were sex differences in the cross-lagged associations between self-efficacy and depressive symptoms. In addition, the study was conducted in a large sample of adolescents and comprised a time span of 2.5 years.

Despite its strengths, the current study also has its limitations. Compared to the Dutch adolescent population, the current sample was comprised of a somewhat smaller percentage of adolescents following lower educational tracks, adolescents from ethnic minority groups, and adolescents living in rural areas. Therefore, generalizing the results to the entire Dutch adolescent population should be done with caution. In addition, the current study was part of a prevention study. However, to take this into account, the intervention condition was included as a covariate as was done in other studies as well (Ringlever et al. 2013; Waller et al. 2012). A second limitation is the fact that we only used self-reports of adolescents’ depressive symptoms and self-efficacy levels. Although self-reports of depressive symptoms are a valid and widely used method (Kovacs 2001), including observations or clinical interviews could have provided a more complete picture. Another limitation was the time span of 6 months between assessments. Since self-efficacy levels showed moderate stability over time during early to middle adolescence in the current study, this could either suggest that self-efficacy is still developing during adolescence or that self-efficacy is state dependent. Therefore, the nature of the associations between depressive symptoms and self-efficacy domains could have been different had the study adopted shorter time spans, e.g., months or weeks.

### Future Research

This is, to our knowledge, the first study that assessed the prospective longitudinal and bidirectional association between depressive symptoms and academic, social and emotional self-efficacy in a large adolescent sample. Therefore, it is important that more studies will be conducted that examine these associations and test whether sex differences exist. These studies should also include shorter time intervals such as months or weeks, to capture the changes in self-efficacy levels that can occur within 6 months time lags. To improve our understanding of the development of depressive symptoms, future research could test hypotheses in which factors from different levels interact, i.e., cognitions, genetics, environment, affect, negative life experiences, as suggested by the cognitive vulnerability-transactional stress model (Hankin and Abramson 2001).

## Conclusion

The present study was the first to examine bidirectional associations between depressive symptoms and academic, social and emotional self-efficacy in a large adolescent population over a time span of 2.5 years, ranging from 14 to 16.5 years. It can be concluded that overall, for both boys and girls, depressive symptoms consistently predicted levels of academic and emotional self-efficacy 6 months later. Self-efficacy on the other hand did not predict subsequent levels of depressive symptoms. These results seem to suggest that depressive feelings have this sort of contaminating effect, negatively impacting other domains of functioning (i.e., academic and emotional self-efficacy) but that low levels of self-efficacy do not lead to aggravation of depressive symptoms experienced 6 months later. In addition, the results seem to suggest that depression prevention programs should not focus on academic, social and emotional self-efficacy as mechanisms through which depressive symptoms should decrease. However, as this was one of the first prospective longitudinal studies to investigate the bidirectional associations between adolescents’ depressive symptoms and academic, social and emotional self-efficacy, more research is needed before firm conclusions can be drawn and implications for practice can be provided. Adolescence is a developmental period in which rapid changes take place, adolescents’ self-efficacy is still developing and might also depend on the events that adolescents encounter, as was found for adults (Yeo and Neal 2006). Future research should therefore be conducted using short and long time spans in order to improve our understanding of how depressive symptoms and self-efficacy mutually influence each other over time.

## Appendix

**Table 2:** Final model: Cross-lagged associations, stability estimates and concurrent correlations of depressive symptoms, academic, social, and emotional self-efficacy

	T2 ← T1	T3 ← T2	T4 ← T3	T5 ← T4	
Cross-lagged associations					
ASE ← SSE (f)	−.023	−.029	−.025	−.031	
ASE ← ESE (f)	.005	.006	.005	.006	
SSE ← ESE (f)	.066	.074	.064	.067	
SSE ← ASE (f)	−.003	−.004	−.003	−.004	
ESE ← ASE (f)	−.019	−.022	−.018	−.022	
ESE ← SSE (f)	−.010	−.013	−.011	−.013	
Stability estimates					
ASE ← ASE (f)	.137***	.157***	.138***	.159***	
SSE ← SSE (f)	.091**	.113***	.093**	.119**	
ESE ← ESE (f)	.128***	.143***	.121***	.146***	
Concurrent correlations	T1	T2	T3	T4	T5
DS—ASE	−.492***	−.327***	−.287***	−.300***	−.354***
DS—SSE	−.357***	−.319***	−.275***	−.328***	−.377***
DS—ESE	−.527***	−.344***	−.292***	−.307***	−.396***
ASE—SSE	.267***	.647***	.571***	.737***	.604***
ASE—ESE	.256***	.526***	.375***	.634***	.470***
SSE—ESE	.354***	.664***	.591***	.774***	.605***

*DS* depressive symptoms, *ASE* academic self-efficacy, *SSE* social self-efficacy, *ESE* emotional self-efficacy, *f* fixed

**p* < 0.05; ***p* < 0.01; ****p* < 0.001
